# Silencing of *Glutamine: Fructose-6-Phosphate Aminotransferase* Impairs Growth and Development in *Sogatella furcifera* (Hemiptera: Delphacidae)

**DOI:** 10.3390/biom13101433

**Published:** 2023-09-22

**Authors:** Zhao Wang, Guiyun Long, Huan Zhu, Daochao Jin, Hong Yang, Cao Zhou

**Affiliations:** 1 College of Environment and Life Sciences, Kaili University, Kaili 556011, China; hdwangzhao@126.com (Z.W.); ab1985916@126.com (H.Z.); 2 School of Ethnic-Minority Medicine, Guizhou Minzu University, Guiyang 550025, China; lgy0256@126.com; 3 Provincial Key Laboratory for Agricultural Pest Management of Mountainous Regions and Scientific Observation and Experimental Station of Crop Pests in Guiyang, Ministry of Agriculture and Rural Affairs of the People’s Republic of China, Institute of Entomology, Guizhou University, Guiyang 550025, China; 4 College of Life Sciences, Chongqing Normal University, Chongqing 401331, China; zhouc@cqnu.edu.cn

**Keywords:** glutamine: fructose-6-phosphate aminotransferase, gene expression, RNA interference, *Sogatella furcifera*

## Abstract

Glutamine: fructose-6-phosphate aminotransferase (GFAT), the fourth enzyme in the chitin synthesis pathway, exerts wide-ranging effects on the growth and development of organisms. However, the role of *GFAT* in *Sogatella furcifera* remains unknown. In this study, the functional significance of the *GFAT* gene of *S. furcifera* was analyzed using a reverse transcription-polymerase chain reaction and RNA interference (RNAi) analyses. The complementary DNA sequence of *SfGFAT* was 3162 bp in length and contained a 2067 bp open reading frame encoding 688 amino acid residues. Structural domain analysis indicated that the *Sf*GFAT protein consisted of one glutamine aminotransferase class 2 domain and two sugar isomerase domains. Expression profile analysis revealed that *SfGFAT* was expressed throughout the egg, nymph, and adult phases and was strongly expressed on the first day of each nymph stage and in the integuments of five tissues. RNAi results revealed that *SfGFAT* gene silencing significantly inhibited the mRNA expression of the target gene and resulted in severe mortality among *S. furcifera*. In summary, these findings demonstrate that *SfGFAT* plays a critical role in the development of *S. furcifera*. Moreover, these results may aid in the development of methods to control the spread of *S. furcifera*.

## 1. Introduction

*Sogatella furcifera* (Horváth) (Hemiptera: Delphacidae) is a typical hemimetabolous rice pest found in many East Asian countries [1,2,3]. It is known to damage rice through sucking sap directly from the phloem of rice plants and through ovipositing on rice [4,5,6,7]. Moreover, it is known to transmit rice viruses, such as southern rice black-streaked dwarf virus. Once rice is infected with the virus, it can cause severe stunting and reduce the setting rate, thus leading to rice yield losses [8,9,10]. The growth and development of *S. furcifera* progresses through three sequential stages: egg, nymph, and adult stages. Moreover, the nymph stage is divided into five instars. Thus, *S. furcifera* must undergo five molting processes to develop from a nymph to an adult [11,12,13,14]. Molting and wing expansion are therefore the key processes for the growth and migration of *S. furcifera*. In insects, both molting and wing development require the production of chitin [15,16,17].

Chitin, a linear polysaccharide homopolymer of *N*-acetylglucosamines, is an essential structural composition of the insect cuticle and therefore a pivotal target for controlling pest insects [18,19]. Chitin biosynthesis in insects is a complex and dynamic process involving at least eight enzymes [20,21]. Chitin synthesis begins with the hydrolysis of trehalose to glucose by trehalase (Tre). Next, glucose enters a sequential catalysis reaction involving hexokinase (HK), glucose-6-phosphate isomerase (G6PI), glutamine: fructose-6-phosphate aminotransferase (GFAT), glucosamine-6-phosphate *N*-acetyltransferase (GNA), phosphoacetylglucosamine mutase (PAGM), and UDP-*N*-acetylglucosamine pyrophosphorylase (UAP). Finally, a chitin polymer is synthesized by chitin synthase (CHS) [22]. To date, only three of the enzymes involved in the chitin synthesis pathway (i.e., Tre, UAP, and CHS) have been widely studied [23,24,25,26,27]. In contrast, data regarding the molecular and functional characterization of other chitin synthetic enzymes in insects are limited.

GFAT (EC 2.6.1.16), a pivotal rate-limiting enzyme in the hexosamine pathway, specifically catalyzes the conversion of fructose-6-phosphate and glutamine into glucosamine-6-phosphate [28]. This reaction product is then further processed by several enzymes to produce uridine diphosphate-*N*-acetylglucosamine, a vital precursor molecule for chitin synthesis in insects [29]. To date, GFAT has been identified in relatively few insect species, including *Drosophila melanogaster* [30], *Aedes aegypti* [31], *Nilaparvata lugens* [32], and *Hyphantria cunea* [33]. In *D. melanogaster*, GFAT activity can be inhibited by UDP-*N*-acetylglucosamine, which acts to regulate the rate of chitin formation [30]. In *A. aegypti*, *GFAT* expression is upregulated in the midgut after blood feeding, and RNAi knockdown of *AeGFAT-1* was found to severely impair the formation of a peritrophic matrix [31,34]. Furthermore, the silencing of *GFAT* in *N. lugens* led to a decrease in the expression of genes related to chitin metabolism and caused very high levels of malformation and mortality [32]. In addition, knockdown of *GFAT* caused the downregulation of other genes, including *GNA*, *PAGM*, *UAP*, and *CHSA*, thereby resulting in decreased chitin content in the epidermis [33]. Overall, data from previous studies suggest that *GFAT* plays an essential role in the regulation of insect growth and metamorphosis.

In this research, a *GFAT* gene (*SfGFAT*) was identified for the first time in *S. furcifera*. In addition, structural molecular characteristics and a phylogenetic tree including *SfGFAT* were determined via bioinformatic analyses. Moreover, the biological function of *SfGFAT* was determined using RNA interference (RNAi) knockdown. These results can help us understand the biological function of *SfGFAT* in planthopper chitin synthesis and may provide a potential target for the development of new chitin synthesis inhibitors.

## 2. Materials and Methods

### 2.1. Insect Rearing and Sample Collection

The *S. furcifera* were raised in a growth chamber at the institute of entomology, Guizhou University, Guiyang, China. The insects rearing was kept in mesh cages with fresh Taichung Native-1 rice seedlings at 25 °C ± 1 °C, 70% ± 10% relative humidity, and a 16:8 h (L:D) photoperiod [15].

As described previously [35], samples were collected during the feeding process. The sample collection schedule included 18 time points from the egg stage to the adult stage, including 1–2-day-old egg (EG1–EG2), 1–2-day-old 1st instar nymph (1L1–1L2), 1–2-day-old 2nd instar nymph (2L1–2L2), 1–3-day-old 3rd instar nymph (3L1–3L3), 1–3-day-old 4th instar nymph (4L1–4L3), 1–3-day-old 5th instar nymph (5L1–5L3), and 1–3-day-old adult (AD1–AD3). Samples of different tissues were collected from the head, integument, fat body, and gut of 1-day-old 5th instar nymphs and from the ovary of 3-day-old adults. During sampling, three biological replicates of each sample were fleetly frozen in liquid nitrogen and stored at −80 °C prior to use.

### 2.2. Primer Design

Primers were generated based on transcriptome sequencing data of *S. furcifera* (SRR116252). Primer design was performed using Primer Premier version 6.0 (Palo Alto, CA, USA). The sequences of all primers used in this study are shown in Table 1. All primers were synthesized by Sangon Biotech Co., Ltd. (Shanghai, China).

### 2.3. RNA Isolation and cDNA Synthesis

Whole bodies of *S. furcifera* nymphs or adults were used to isolate total RNA for the cloning of *SfGFAT*. First, total RNA was extracted using an HP Total RNA Kit (Omega Bio-Tek, Norcross, GA, USA) with genomic DNA removal columns following the manufacturer’s instructions. The integrity of the extracted RNA was verified via 1% agarose gel electrophoresis. Further analysis using a Nanodrop 2000 spectrophotometer (Thermo Fisher Scientific, Wilmington, DE, USA) was performed to estimate the concentration and purity of RNA. The purified RNA was then stored at −80 °C for future use. An AMV First Strand cDNA Synthesis Kit with an oligo(dT) primer (Sangon Biotech, Shanghai, China) was used to synthesize first-strand complementary DNA (cDNA) following the manufacturer’s instructions. All cDNA samples were then stored at −20 °C for future experiments.

### 2.4. Cloning of SfGFAT

Based on the results of transcriptome sequencing of *S. furcifera* (SRR116252), two cDNA fragments encoding *GFAT* were obtained using Geneious 2020.0.5 (Biomatters, Inc., Auckland, New Zealand). We then amplified these sequences by polymerase chain reaction (PCR) using prosynthetic cDNA and gene-specific primers (GSPs, Table 1). PCR was performed using a Bio-Rad T100 Thermal Cycler PCR System (Bio-Rad, Hercules, CA, USA). In brief, 25 μL reaction mixtures contained 2 μL dNTP (2.5 mM), 2.5 μL 10× LA PCR Buffer (Mg2+ plus), 1 μL of each primer (10 mM), l μL cDNA template, 0.25 μL LA Taq polymerase (TaKaRa, Dalian, China), and double-distilled water up to 25 μL. PCR reaction conditions were as follows: one cycle of pre-denaturation at 94 °C for 3 min; followed by 30 cycles of denaturation at 94 °C for 30 s, annealing at 53 °C for 30 s, and extension at 72 °C for 2 min; and a final extension at 72 °C for 10 min. Finally, the amplified products were examined by 1% agarose gel electrophoresis. The target bands for the desired products were then purified using an EasyPure^®^ Quick Gel Extraction Kit (TransGen Biotech, Beijing, China). The purified DNA was then ligated to a pMD18-T vector (TaKaRa, Dalian, China) and sequenced by Sangon Biotech (Shanghai, China).

Next, we amplified the ends of *SfGFAT* via rapid amplification of cDNA end PCR (RACE-PCR) using a SMARTer RACE 5′/3′ Kit (Clontech, Mountain View, CA, USA). In particular, we used long universal primers and GSPs to perform the primary RACE-PCR. Here, the reaction conditions were as follows: 30 cycles of denaturation at 94 °C for 30 s, annealing at 55 °C–57 °C (according to the primer annealing temperature) for 30 s, and final extension at 72 °C for 60 s. For the nested RACE-PCR reaction, the primary PCR product was initially diluted 100 times before being used as a template with a short universal primer and GSPs. The reaction conditions were the same as those used for the primary PCR reaction. All RACE-PCR products were then purified and sequenced as previously described.

### 2.5. Bioinformatics Analyses

All obtained sequencing fragments were assembled using SeqMan version 5.0 (DNASTAR, Inc., Madison, WI, USA). The nucleotide sequence was first edited using DNAMAN version 7.0 (Lynnon Biosoft, California, CA, USA). NCBI BLAST (https://blast.ncbi.nlm.nih.gov/Blast.cgi, accessed on 20 August 2019) was used to align homologous sequences. The NCBI Open Reading Frame (ORF) Finder (https://www.ncbi.nlm.nih.gov/orffinder/, accessed on 20 August 2019) was used to predict ORFs present in *SfGFAT*. The physical and chemical properties of the deduced protein were then analyzed using the ExPASy ProtParam tool (https://web.expasy.org/protparam/, accessed on 22 August 2019), SignalP version 4.1 (http://www.cbs.dtu.dk/services/SignalP/, accessed on 22 August 2019), NetNGlyc version 1.0 (https://services.healthtech.dtu.dk/service.php?NetNGlyc-1.0, accessed on 22 August 2019), and TMHMM version 2.0 (https://services.healthtech.dtu.dk/service.php?TMHMM-2.0, accessed on 22 August 2019). A three-dimensional (3D) model of *SfGFAT* was predicted using SWISS-MODEL (https://www.swissmodel.expasy.org/interactive, accessed on 5 September 2019), and this was then visualized using PyMOL Molecular Graphics System version 1.1 (DeLano Scientific LLC, San Carlos, CA, USA). Finally, a neighbor-joining tree was constructed using MEGA version 6.06, and bootstrap analyses of 1000 replicates were performed.

### 2.6. Real-Time Quantitative PCR (RT-qPCR) Analysis of SfGFAT Expression Levels

All primers used for RT-qPCR analyses were designed using Primer Premier version 6.0 and are listed in Table 1. *S. furcifera 18S rRNA* was used as an internal reference gene. RT-qPCR was performed in a CFX-96 real-time quantitative PCR system (Bio-Rad, Hercules, CA, USA) with 20 μL mixtures containing 10 μL FastStart Essential DNA Green Master (Roche, Diagnostics, Shanghai, China), 1 μL (10 μM) of each primer, 1 μL cDNA, and 7 μL hyper-pure water. The PCR amplification conditions were as follows: pre-denaturation at 95 °C for 10 min, 40 cycles of 95 °C for 30 s, and annealing at 55 °C for 30 s. After the reaction, a melting curve analysis was performed from 60 °C to 95 °C to verify the specificity of RT-qPCR products. The relative expression levels of *SfGFAT* were then calculated using the 2^−ΔΔCt^ method [36].

### 2.7. Functional Analysis of SfGFAT Using RNAi

To investigate the biological functions of *SfGFAT*, we used unique primers for *SfGFAT* and added a T7 RNA polymerase promoter (Table 1) for dsRNA synthesis. Templates for in vitro transcription reactions were synthesized by PCR from a plasmid containing *SfGFAT* DNA using these primers. The PCR products were then subcloned and sequenced to ascertain its specificity. Next, the expected fragments were purified using an EasyPure^®^ Quick Gel Extraction Kit (TransGen Biotech, Beijing, China). The concentrations of the purified products were determined using a NanoDrop 2000 spectrophotometer (Thermo Fisher Scientific, Wilmington, DE, USA), and the products were then used for in vitro transcription reactions.

Double-stranded RNA (dsRNA) was synthesized using a MEGAscript^®^ RNAi Kit (Ambion, Carlsbad, CA, USA) with all procedures performed according to the user manual. In vivo *SfGFAT* gene silencing in *S. furcifera* nymphs was conducted as previously described [15,35,37]. Briefly, first day 5th instar nymphs were anesthetized with CO_2_ for approximately 90 s, then placed on a 1% agarose gel plate with grooves. A Nanoliter 2010 Injector (World Precision Instruments, Sarasota, FL, USA) was then used to inject 100 ng of ds*GFAT* into the junction of the prothorax and mesothorax of *S. furcifera* subjects; in some instars, ds*GFP* was injected as a negative control. Each experimental treatment (*n* = 50) contained three biological replicates. Subsequently, injected nymphs were maintained on fresh rice seedlings and abnormality and mortality rates were assessed daily. Photographs of abnormal insects were captured using a Keyence VH-Z20R stereoscopic microscope (Keyence, Osaka, Japan). In addition, 10 injected insects were selected randomly at 72 h after injection for evaluation of their mRNA levels. The RNAi efficiency was then evaluated via RT-qPCR performed using primers listed in Table 1.

### 2.8. Statistical Analysis

All data were statistically analyzed using Microsoft Excel 2003 and SPSS version 13 (IBM SPSS Inc., Chicago, IL, USA). The relative expression of *SfGFAT* at different stages and in different tissues of *S. furcifera* was determined using the 2^−ΔΔCt^ method. All data were expressed as the mean ± standard error (SE) of three replicates. Differences in gene expression at different stages and in different tissues were calculated using one-way analysis of variance (ANOVA) followed by Duncan’s multiple range test (*p* < 0.05). Finally, an independent sample *t*-test was used to evaluate the statistical significance of gene silencing.

## 3. Results

### 3.1. Identification and Sequence Analysis of SfGFAT

**Figure 1:**
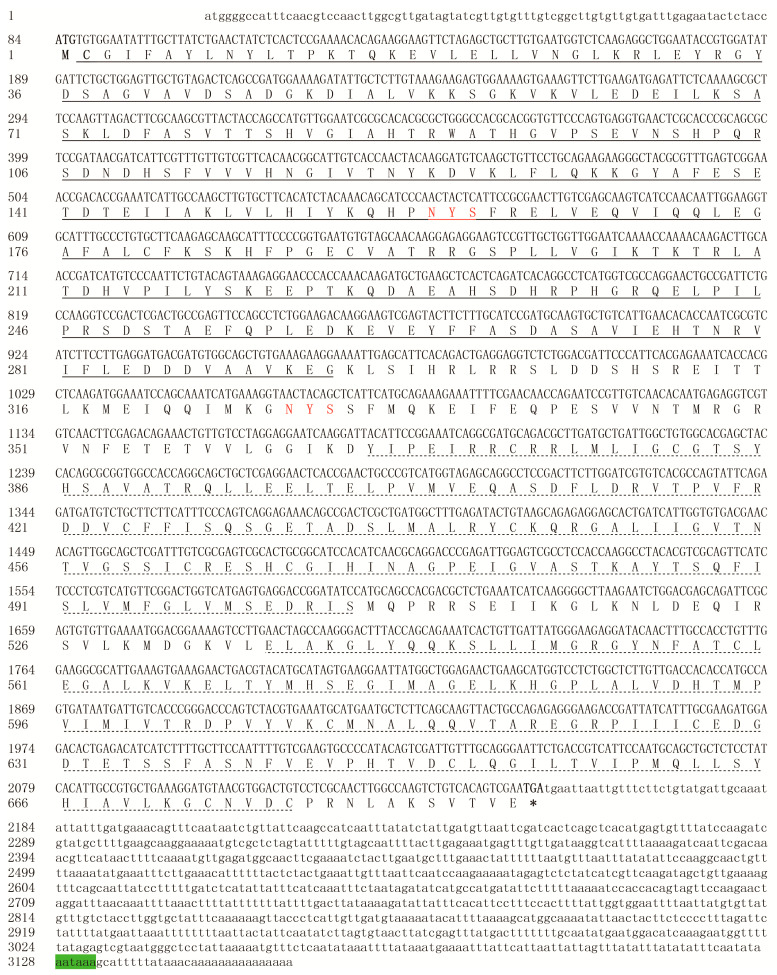
Nucleotide sequence of *SfGFAT* cDNA and deduced amino acid sequence. The start codon is highlighted in bold, and the stop codon is indicated in bold with an asterisk. The two putative *N*-glycosylation sites are shown in red. The polyadenylation signal is shadowed in green. Moreover, the glutamine aminotransferase class 2 domain is underlined, and the sugar isomerase domains are indicated with dotted lines.

The entire cDNA sequence of *SfGFAT* was identified from the DNA fragments amplified using PCR and 5′/3′ RACE (GenBank registration number: MF964939). The *SfGFAT* sequence was 3162 bp long and included a 5′ noncoding region of 83 bp and a 3′ noncoding region of 1012 bp. The ORF of *SfGFAT* was 2067 bp long and encoded 688 amino acid residues. The 3′ end of the cDNA sequence of *SfGFAT* contained a typical AATAAA tail and a poly-A structure (Figure 1). The theoretical molecular weight of this protein was 76.89 kDa, and its theoretical isoelectric point (pI) value was 6.33. Next, we used the NetNGlyc version 1.0 Server to predict potential *N*-glycosylation sites and found two sites at residues 159 and 327 (Figure 1). Further analysis revealed that the SfGFAT protein did not contain signal peptide and transmembrane helices.

**Figure 2:**
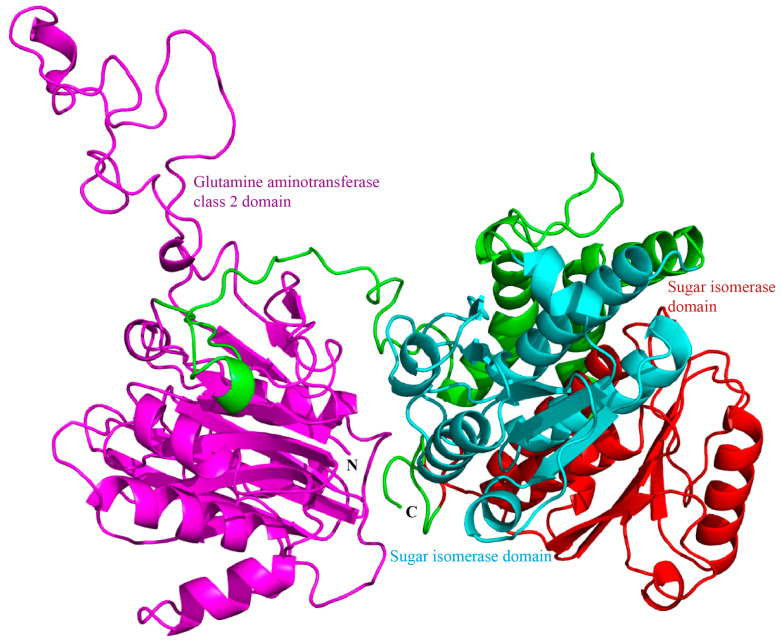
Three-dimensional model of *Sf*GFAT.

We then used the SWISS-MODEL online tool to model the homology of *Sf*GFAT. We found that the *Sf*GFAT protein consisted of three domains (Figure 2). These included a glutamine aminotransferase class 2 domain (GAT2) in the N-terminus of the protein, which may be responsible for catalyzing the transfer of an amino group from glutamine, as well as two sugar isomerase domains (SIS) in the C-terminus, which function as phosphosugar isomerases or phosphosugar binding proteins.

**Figure 3:**
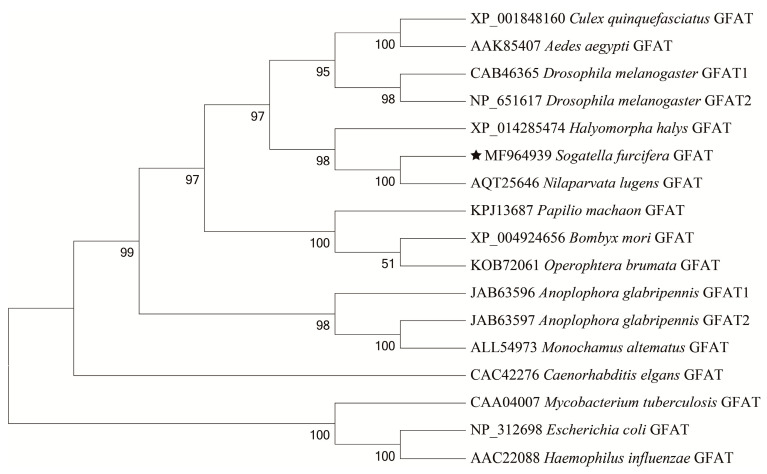
Phylogenetic analysis of GFAT proteins from *S. furcifera* and other organisms.

We then used BLAST to query for homologous sequences of the amino acid sequence encoded by *SfGFAT*. These results revealed that the amino acid sequence of *Sf*GFAT shared the highest identity with the Hemipteran *N. lugens* (KU556833.1, 89.63% identity) followed by the Lepidopteran *Plutella xylostella* (XM_011570168.3, 74% identity). To explore the evolutionary relationships of GFAT and homologous proteins, a phylogenetic tree was constructed using the neighbor-joining method as implemented in MEGA version 6.06 (Figure 3). The tree indicated that *Sf*GFAT has a close evolutionary relationship with other Hemipteran insects, especially *N. lugens*.

### 3.2. Spatiotemporal Expression Profile of SfGFAT in S. furcifera

**Figure 4:**
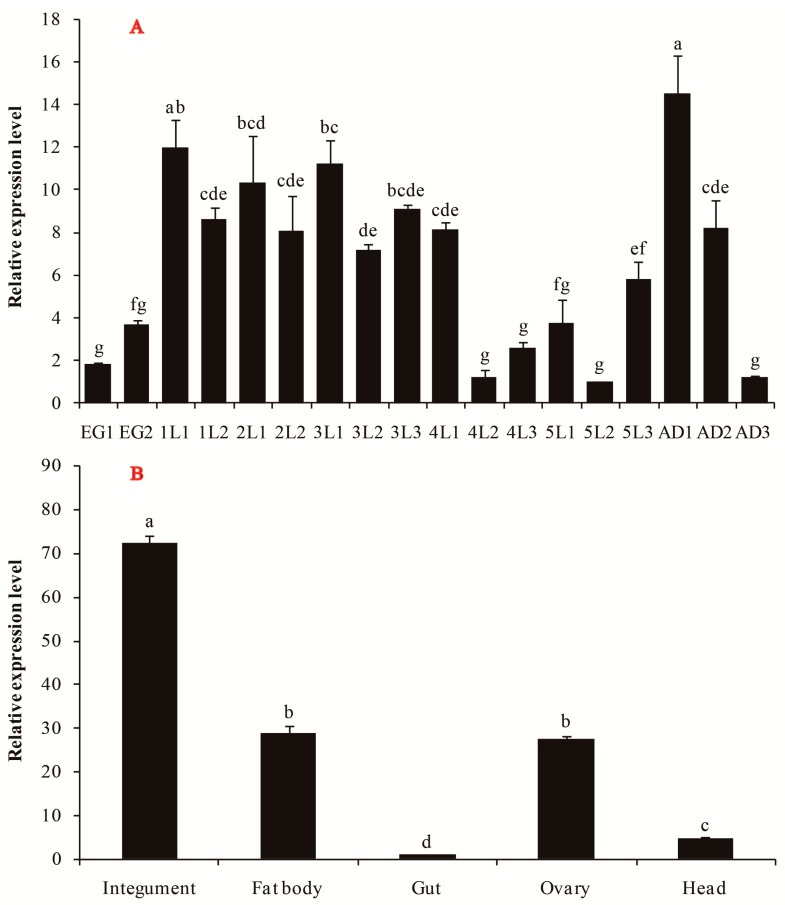
Developmental stages (**A**) and tissues (**B**) mRNA levels of *SfGFAT* in *S. furcifera*. Relative mRNA levels of *SfGFAT* were measured using qRT-PCR. Data were normalized using *S. furcifera* 18S rRNA and are shown as the mean ± SE of three independent tests. Different letters imply statistically significant differences in mean expression (*p* < 0.05, Duncan’s multiple range test in one-way ANOVA).

Next, we examined the spatiotemporal expression profiles of *SfGFAT* at various developmental stages ranging from the egg to adult stages using RT-qPCR. We observed that *SfGFAT* was continuously expressed across the 18 examined developmental points. Moreover, the relative *SfGFAT* mRNA expression levels were found to increase just before molting days, reach their highest levels immediately after molting, and then decrease afterward. Finally, peak *SfGFAT* mRNA expression was found to occur on the first day of adulthood (Figure 4A).

Next, we determined the expression levels of *SfGFAT* for the integument, fat body, gut, head, and ovary (Figure 4B). *SfGFAT* expression was significantly higher in the integument than in the other tissues followed by the fat body and ovary. The lowest level of *SfGFAT* expression was detected in the gut. The relative expression level of *SfGFAT* in the integument was 72.38, which was 2.51, 2.63, and 14.77 times higher than that in the fat body, ovary, and head, respectively.

### 3.3. Functional Analysis of SfGFAT

#### 3.3.1. Analysis of *Sf*GFAT mRNA Levels and Survival after RNAi Exposure

**Figure 5:**
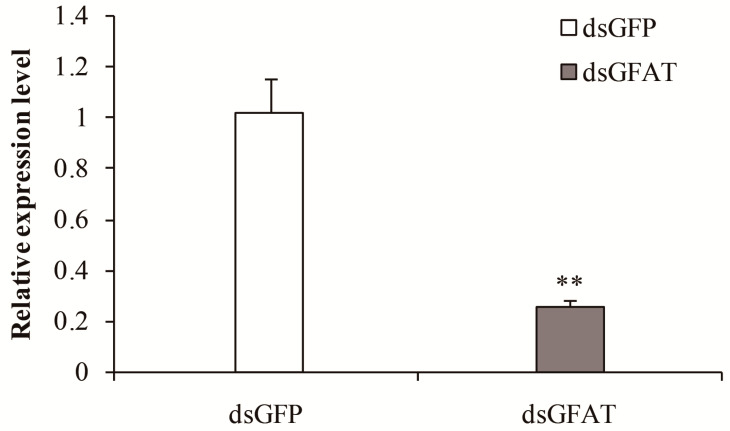
Relative mRNA levels of *SfGFAT* after injection with an interfering dsRNA (dsGFAT) and a negative control (dsGFP). Values are shown as the mean ± SE of three independent tests. ** indicates extremely significant differences (*p* < 0.01).

To explore the functional significance of *SfGFAT*, dsRNAs prepared in vitro were injected into newly molted fifth instar nymphs. After 72 h, we collected surviving insects and determined the mRNA levels of *SfGFAT* present (Figure 5). RT-qPCR results indicated that the levels of *SfGFAT* mRNA were significantly inhibited following dsGFAT injection (*p* < 0.01). 

**Figure 6:**
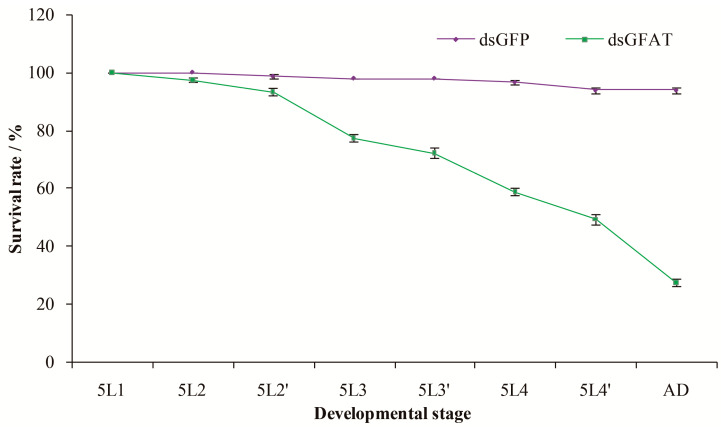
Survival rates of *S. furcifera* after dsRNA injection. Shown are the survival rates of insects injected with 100 ng dsGFAT and dsGFP dsRNAs on the first day of fifth instar nymphs. Insect age in days is displayed on the X-axis; e.g., 5L1, first day of fifth instar nymphs; 5L2 and 5L2′ represent the two 12 h halves of a single day; AD, adults. Values show the mean ± SE of three independent tests.

The survival rates of the tested *S. furcifera* individuals were continuously monitored following injection to ascertain whether their development was altered in response to *SfGFAT* gene silencing (Figure 6). These results showed that the cumulative survival rate declined gradually over time. In particular, no change in survival between the dsGFAT and dsGFP groups 12 h after injection was detected. However, from 24 h onward, the survival of individuals injected with dsGFAT decreased sharply, with only 49.3% of the individuals surviving to eclosion. After eclosion, the survival rate was only 27.3%.

#### 3.3.2. Phenotype Analysis after RNAi

**Figure 7:**
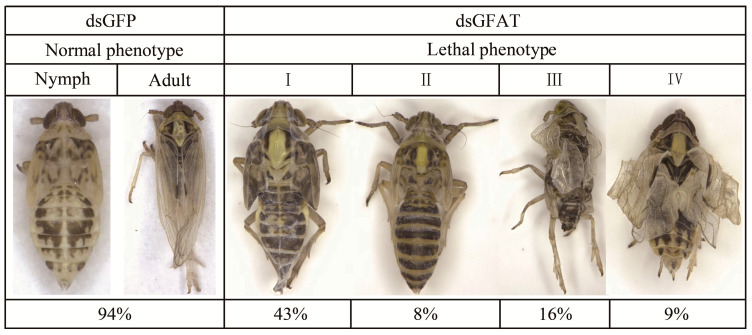
Lethal phenotypes caused by *SfGFAT* RNAi.

After the successful injection of dsGFAT, the tested insects exhibited four different lethal phenotypes (Figure 7). Approximately 43% of the malformed individuals exhibited a “double-skin” phenotype (I); 8% displayed a “wasp-waisted” phenotype in which the body of the injected nymphs was significantly longer, and the junction between the chest and abdomen was narrower (II); 16% did not shed their old cuticle normally and showed shrunken wings (III); and 9% with a smaller body and misshaped wings died (IV).

## 4. Discussion

Chitin is the second most abundant biological polysaccharide matrix and provides structural support to the insect exoskeleton, tracheal system, and alimentary canal [38]. Insect chitin remodeling is a highly complex process that is regulated by several enzymes. Previous studies have suggested that the suppression of specific insect chitin remodeling enzymes may be a useful strategy for developing pest control treatments [23,35,37,39]. GFAT is a crucial enzyme that catalyzes the rate-limiting step of the chitin biosynthesis pathway. However, the molecular mechanisms and functions of *GFAT* underlying the regulation of chitin biosynthesis in *S. furcifera* remain unknown.

In the present study, we characterized *SfGFAT*, the *GFAT* gene from *S. furcifera*. Our results revealed that the *SfGFAT* cDNA sequence was 3162 bp in length and encoded a protein containing 688 amino acids. A previous study on *A. aegypti* showed that the sequence of its *GFAT* gene contained a 3′ noncoding region that was 770 bp in length [31]. This finding is consistent with the findings of our study since we found that *SfGFAT* also contains a long 3′ noncoding region (i.e., even longer than *AeGfat-1*), suggesting the possibility of complex regulation on the translation level. Next, BLAST analysis revealed that the *N. lugens* GFAT shared 89.63% identity with the *S. furcifera* GFAT. Phylogenetic analysis indicated that the GFATs of *S. furcifera* and the hemipteran *N. lugens* were more closely related than *SfGFAT* and the GFATs of other eukaryotes and bacteria. Furthermore, structural domain analysis showed that *Sf*GFAT contained a GAT2 domain and two SIS domains, which is similar to the structures of other previously described GFATs. The N-terminal glutamine aminotransferase class 2 domain hydrolyzes glutamine to release an amino group, which then transfers to a new substrate. In addition, the two C-terminal sugar isomerase domains are involved in phosphosugar isomerization and are able to bind phosphosugars [30,31,40,41,42].

Based on the observed mRNA levels of *SfGFAT* at different developmental stages, it is noteworthy that *SfGFAT* expression is periodically upregulated just before each molting cycle. More specifically, the relative mRNA level of *SfGFAT* increased significantly just before the molting days, reached its highest expression level immediately after each molting, and decreased thereafter. One reasonable explanation for this phenomenon is that the formation and hardening of the new cuticle of *S. furcifera* requires a large amount of chitin and therefore an active chitin biosynthesis pathway. Moreover, the expression trends observed for *SfGFAT* are highly analogous to trends found in our previous studies of *S. furcifera* [15,35,37]. In addition, a recent study of *N. lugens* revealed that *NlGFAT* was continuously expressed in all developmental stages after the fourth instar and also showed relatively higher expression levels during molting [32]. Previous studies have also verified that the expression of *GFAT* is tissue-specific. In *N. lugens*, *GFAT* was widely detected in a variety of tissues, but *NlGFAT* was most highly expressed in the wing bud and cuticle [32]. Our tissue-specific expression experiment indicates that *SfGFAT* was also ubiquitously expressed in the tissues of *S. furcifera* but showed the highest mRNA levels in the integument, which contain a great deal of chitin. This finding is therefore consistent with the hypothesis that *GFAT* expression is closely linked to chitin biosynthesis. Similarly, a *GFAT* in *Haemaphysalis longicornis* was found to be present in various tissues, including the cuticle, midgut, salivary gland, and ovary [40]. This was consistent with our finding that *SfGFAT* showed relatively high expression in the ovary. Another previous study of *A. aegypti* also demonstrated that chitin material is present in the ovaries [43]. We therefore speculate that *SfGFAT* plays an essential role in chitin biosynthesis and insect reproduction.

Since chitin biosynthesis and degradation pathways are unique to insects, they have been identified as potential targets for controlling pest populations using RNAi methods [44]. Knockdown of chitin metabolic pathway genes using RNAi has been reported to be a practical method for controlling planthoppers. For example, ds*CHS1* injection causes a significant decrease in the transcript levels of *CHS1* and a remarkable increase in the malformation rates and mortality of both *S. furcifera* and *N. lugens* [15,45]. Moreover, the knockdown of *N. lugens Tre* was found to inhibit the relative expression of other genes involved in the chitin metabolic pathway (e.g., *HK*, *G6PI*, chitinase, and *CHS*) and cause severe molting deformities and mortality [46]. In addition, ds*NlTPS* injection can reduce the mRNA levels of *TPS* and thereby induce a lethal response in *N. lugens* nymphs [47]. Similarly, RNAi-mediated downregulation of *SfUAP* has been found to seriously affect the growth and metamorphosis of *S. furcifera* [37]. In our experiment, the transcript levels of *SfGFAT* significantly decreased following RNAi injection. After eclosion, the survival rate decreased to 27.3%, which further indicated that dsRNA successfully suppressed the expression of *GFAT*. Finally, dsGFAT injection led to a significant increase in abnormality rates and lethality rates. These findings were consistent with those obtained in *N. lugens* [32].

## 5. Conclusions

In summary, we successfully identified the *GFAT* cDNA of *S. furcifera* and assessed the normal dynamic changes induced by *SfGFAT* at several different developmental stages and in several different tissues. Moreover, using RNAi, we found that the knockdown of *SfGFAT* severely inhibited the expression of the target gene and caused severe molting difficulty and wing malformation in *S. furcifera*. Overall, our findings demonstrate that *SfGFAT* plays a vital role in chitin synthesis as well as indicate that *SfGFAT* may serve as a promising candidate gene for future planthopper control treatments.

## Figures and Tables

**Table 1:** Primer sequence information.

Gene	Notes	Primer Name	Primer Sequence (5′–3′)
*SfGFAT*	*SfGFAT*	SfGFAT-F	CGAGCAAGTCATCCAACA
	cloning	SfGFAT-R	GGTCAACAAGAGCCAGAG
		5′GFAT-R1	TTTGGTGGGTTCCTCTTTAC
		5′GFAT-R2	ACTTCCTCTCCTTGTTGCT
		3′GFAT-F1	TGCCAGTGATAATGATTGTC
		3′GFAT-F2	GAAGATGGAGACACTGAGAC
	RT-qPCR	qGFAT-F	CGAAGATGGAGACACTGAG
	for *SfGFAT*	qGFAT-R	CGGCAATGTGATAGGAGAG
	ds*GFAT*	dsGFAT-F	TAATACGACTCACTATAGGGGTAGCAACAAGGAGAGGAAG
	synthesis	dsGFAT-R	TAATACGACTCACTATAGGGACAGCCAATCAGCATCAAG
*Sf18S*	RT-qPCR for	q18S-F	CGGAAGGATTGACAGATTGAT
rRNA	reference gene	q18S-R	CACGATTGCTGATACCACATAC
GFP	ds*GFP*	dsGFP-F	TAATACGACTCACTATAGGGAAGGGCGAGGAGCTGTTCACCG
	synthesis	dsGFP-R	TAATACGACTCACTATAGGGCAGCAGGACCATGTGATCGCGC

Note: The underlined sequence represents the T7 promoter.

## Data Availability

The data were deposited in GenBank under accession number MF964939.
